# Decreased Intra- and Inter-Salience Network Functional Connectivity is Related to Trait Anxiety in Adolescents

**DOI:** 10.3389/fnbeh.2015.00350

**Published:** 2016-01-21

**Authors:** Haiyang Geng, Xuebing Li, Jie Chen, Xinying Li, Ruolei Gu

**Affiliations:** ^1^Key Laboratory of Mental Health, Institute of Psychology, Chinese Academy of SciencesBeijing, China; ^2^State Key Laboratory of Cognitive Neuroscience and Learning, Beijing Normal UniversityBeijing, China; ^3^Key Laboratory of Behavioral Science, Institute of Psychology, Chinese Academy of SciencesBeijing, China

**Keywords:** anxiety, salience network (SN), anterior insula (AI), dorsal anterior cingulate cortex (dACC), resting-state fMRI

## Abstract

**Objective:** Adolescence is a critical period for the vulnerability of anxiety. Imaging studies focusing on adolescents' susceptibility to anxiety suggest that the different development trajectories between the limbic system and the executive control system may play important roles in this phenomenon. However, few studies have explored the brain basis of this susceptibility from the perspective of functional networks. The salience network (SN) consists of a series of key limbic and prefrontal regions that are engaged in the development of anxiety, such as the amygdala, anterior insula (AI), and dorsal anterior cingulate cortex (dACC). Intra- and inter-network connections in this system play essential roles in bottom-up attention and top-down regulation of anxiety, nevertheless, little is known about whether the SN-centered connections are associated with trait anxiety (i.e., susceptibility to anxiety) in adolescents.

**Method:** Here, we applied resting-state functional magnetic resonance imaging (fMRI) to explore the relationship between intra- and inter-network functional connectivity (FC) of the SN and trait anxiety in adolescents using the amygdala, AI and dACC as the regions of interest (ROI).

**Results:** We found that trait anxiety levels were inversely associated with both characteristic AI-dACC FC in the SN and distributed inter-network FC between the SN and multiple functional systems, which included the default mode network and the executive control network.

**Conclusions:** Our results indicate that weaker intra- and inter-network FC of the SN was linked to higher trait anxiety among adolescents, and it may underlie altered salience processing and cognitive regulation.

## Introduction

The classical neurocognitive model of anxiety proposes that the disruption of the amygdala-prefrontal circuitry in anxiety, which represents deficient recruitment of prefrontal control and amygdala hyper-responsivity to threat, leads to alterations in salience processing, and cognitive control (Bishop, 2007). These mechanisms are implicated in the maintenance and possibly even the etiology of anxiety (Bishop, 2007). Specific to the adolescent stage, adolescents experience persistent negative, and labile mood states (e.g., anxiety; Somerville et al., 2010). Sustained high anxiety during this period can result in increased vulnerability to anxiety-related disorders (Paus et al., 2008). Although adolescents are highly vulnerable to anxiety (Crone and Dahl, 2012), the neurocognitive underpinning of this vulnerability remains poorly understood.

A neurodevelopmental theory examining this susceptibility, which combines the classical model of anxiety and neurodevelopment evidences, suggests that different developmental trajectories of the amygdala and prefrontal cortex may underlie adolescents' heightened responsiveness to threat and immature engagement of cognitive regulation (Somerville et al., 2010). Aside from the amygdala and prefrontal cortex, the anterior insula (AI) and dorsal anterior cingulate cortex (dACC) are also heavily involved in the core mechanisms of anxiety, such as appraising and regulating emotional salience (Craig, 2009; Etkin et al., 2011; Uddin et al., 2011). The activation of these structures plays a crucial role in the threat-related processing bias found in anxious individuals. However, it is unclear whether interactions between these core regions are related to anxiety.

In our opinion, the salience network (SN), which mainly consists of the amygdala, AI and dACC, may be a candidate for examining the neural basis underlying the vulnerability of anxiety in adolescents. Many brain-imaging studies suggest that the SN plays a central role in detecting emotional salience and triggering cognitive control via its functional connectivity with several distributed brain systems, including the limbic system, primary cortices and the cognitive control network (Seeley et al., 2007; Bressler and Menon, 2010). Moreover, these three regions in this network are individually involved in salience processing and cognitive control, and intra-connections in this network may play distinct roles in these two processes. Amygdala-related circuits are more involved in salience processing (Kim et al., 2011; Baur et al., 2013); in contrast, the AI-dACC circuit mainly triggers dorsal lateral prefrontal cortex (dlPFC)-involved cognitive control (Sridharan et al., 2008; Bressler and Menon, 2010). The amygdala is known to react to emotional and novel stimuli via its connectivity with polymodal associative cortices (LeDoux, 1995), which suggests a crucial role in bottom-up salience processing (Santos et al., 2011). The AI is linked to emotional awareness and subjective feelings generated from the body (Craig, 2010). Signals received from polymodal associative cortices can converge with a higher-order interoceptive representation in the amygdala-AI circuit, which was found to represent state anxiety (Baur et al., 2013). Additionally, activation of the dACC was also found in different types of threat appraisal (Etkin et al., 2011). Two resting-state fMRI studies found that the amygdala-AI and the amygdala-dACC circuits were positively related to state anxiety (Kim et al., 2011; Baur et al., 2013). The authors suggested that stronger connections between these regions reflected an increased sensitivity to salient events, which biased attentional and perceptual processing (Kim et al., 2011; Baur et al., 2013).

Conversely, the AI-dACC circuit in the SN may play a major role in cognitive control instead of simple salience processing. Firstly, the AI and dACC showed reliable activation at the beginning and through sustained periods across multiple tasks, which indicated that the system that includes these two regions may select and modulate the goal-related information at hand (Dosenbach et al., 2006). Furthermore, one Granger causality and latency analysis study found that the AI-dACC network showed directed connectivity to the dlPFC and earlier activation compared to the dlPFC across task paradigms and stimulus modalities. This suggested that the AI-dACC network plays a critical and causal role in triggering the executive control network that includes dlPFC (Sridharan et al., 2008). Critically, two important reviews proposed a central role of dACC in dlPFC-involved cognitive control mechanisms triggered by salience (motivation, conflict, and error) evaluation (Botvinick et al., 2001). In an anxiety-related context, Bishop (2009) proposed that the weaker cognitive control that is indicated by dlPFC activation is a core feature of trait anxiety. Although the activation of the dACC was not found to be involved in Bishop's study, the author suggests that the connection between the dACC and the dlPFC probability plays a role in triggering cognitive control of anxiety (Bishop, 2009). Taken together, the AI-dACC circuit is proposed to be strongly involved in dlPFC-related cognitive control triggered by salience evaluation (Mathews and MacLeod, 1994; Desimone and Duncan, 1995; Mathews and Mackintosh, 1998; Bressler and Menon, 2010), which may be impaired in individuals with anxiety. However, no study has examined the relationship between anxiety and the AI-dACC circuit.

The current study focuses on linking trait anxiety at the individual level with the SN. Trait anxiety is often used as an index of vulnerability to anxiety disorders (Kim and Whalen, 2009; Indovina et al., 2011). Theoretical models and empirical evidence suggest that high trait anxiety and anxiety disorders share common altered activations and connections of SN-related regions (Etkin and Wager, 2007; Sylvester et al., 2012), which are involved in fear, conflict salience, and cognitive control. Therefore, we suggest that trait anxiety be regarded as a candidate to investigate SN-centered networks that underlies the vulnerability to anxiety disorders without the confounding effects of psychotropic medication or chronic illness.

In this experiment, we collected the resting-state imaging data and used the basolateral amygdala (BLA), AI and dACC as regions of interest (ROI) to examine the relationship between the intra-network functional connectivity (FC) of the SN and individual levels of trait anxiety in 60 adolescents. The trait anxiety levels were measured using the trait anxiety scale of Spielberger's State and Trait Anxiety Inventory (STAI-T; Spielberger et al., 1983; Shek, 1993). Voxel-wise resting-state functional connectivity (rsFC) analysis using the same ROIs was employed to detect whether the inter-network FC of the SN with other regions in the whole brain is related to trait anxiety in adolescents. Most importantly, considering the AI-dACC circuit is involved in cognitive control triggered by salience evaluation, we hypothesized that higher levels of trait anxiety in adolescents would be associated with weaker AI-dACC FC, which would indicate impaired cognitive control in trait anxiety. Moreover, considering the amygdala-AI and amygdala-dACC circuits may underlie salience and vigilance in a particular situation, we hypothesized that the FC of these connections would be positively associated with state anxiety compared to trait anxiety. Finally, the SN may have altered inter-network FC with distributed brain regions engaged in emotional processing and cognitive control.

## Methods

### Emotional measurements

Sixty-three healthy participants (Mean ± SD: 15.67 ± 1.00 years, 35 Male/28 Female) were recruited from the local community via media advertisements. They had no history of substance abuse, brain injury, or neurological diseases and no personal or family history of mental disorders, which were measured by an in-house questionnaire. Non-clinical samples were of interest for the questions in the current study because the neural underpinnings of non-psychiatric individuals with trait anxiety may predict those individuals' risk of psychopathology, and they are less likely to be confounded with a disease state. All participants (right-hand) completed the Chinese version of the trait form of Spielberger's State and Trait Anxiety Inventory (STAI-T; Spielberger et al., 1983; Shek, 1993). The STAI-T has been used in multiple studies that investigated anxious characteristics in non-clinical samples, largely due to its sensitivity as a marker of one's risk for anxiety disorders (Grupe and Nitschke, 2013). Many studies suggest that the STAI state scale is highly related to the trait scale and so it is not good enough to dissociate from the trait scale (Spielberger et al., 1983; Shek, 1993); therefore, the Negative Affect subschedule (NAS) of the Positive and Negative Affect Schedule (PANAS) was used to estimate the emotional states of participants before scanning (Watson and Clark, 1999). To control for the effect of depression during analysis, the Children's Depression Inventory (CDI) was used to assess self-reported symptoms of depression (Kovacs, 1985). One participant was excluded because his STAI-T score (25) was lower than the mean value of the sample (M ± SD: 39.63 ± 6.12) minus two standard deviations. This study has been approved by the Ethics Committee of the Institute of Psychology, Chinese Academy of Sciences, and has therefore been performed in accordance with the ethical standards laid down in the 1964 Declaration of Helsinki.

### MRI data acquisition and preprocessing

Experiments were performed in a 3 Tesla SIEMENS MRI scanner (Beijing, China). Functional images were acquired with single-shot gradient-recalled echo planar imaging (GR-EPI) sequences (TR = 2000 ms, TE = 30 ms, FA = 90°, matrix = 64 × 64, FOV = 22 cm, 3-mm slice thickness, 1 mm spacing between slices, 32 transverse slices), aligned along the anterior commissure-posterior commissure (AC-PC) line, and they lasted for 450 s. Subjects were instructed to keep their eyes closed and think of nothing, but to not fall asleep. For spatial normalization, T1-weighted anatomical images were acquired in an axial orientation using a 3D gradient-recalled sequence (TR = 2530 ms, TE = 3.37 ms, FA = 7°, matrix = 256 × 192, 1.33-mm slice thickness) for each subject.

Data preprocessing was performed using DPARSF software (Yan and Zang, 2010, http://www.restfmri.net). The first 10 volumes were discarded to guarantee steady-state longitudinal magnetization. The remaining volumes were then realigned to correct for head motion. Two subjects were excluded because their head movement exceeded ± 3 mm in translation or ± 3° in rotation. Subsequently, realigned volumes were corrected for slice acquisition timing, and then were normalized into a standard stereotaxic space with a resolution of 3 × 3 × 3 mm^3^ using the Montreal Neurological Institute (MNI) echo-planar imaging template in Statistical Parametric Mapping 8 (SPM8; Wellcome Trust Centre for Neuroimaging), which is a free and open source software written in MATLAB (The MathWorks, Inc.). Functional images were spatially smoothed by convolution with an isotropic Gaussian kernel (FWHM = 4 mm). Linear detrend and filtering (0.01–0.08 Hz) were applied. Nuisance signals involving six head motion parameters, cerebrospinal fluid signals, and white matter signals were regressed out.

### Functional ROI definition

Left and right BLA ROIs in the current study were obtained as the result masks from a previous study in the Montreal Neurological Institute (MNI) space (Baur et al., 2013). The authors used maximum probability maps (the threshold is 40%), which allow for the construction of non-overlapping ROIs where each voxel of the amygdala was assigned to one specific subregion on the basis of the maximum probability of belonging to a subgroup (SPM8; Wellcome Trust Centre for Neuroimaging). Furthermore, two AI ROIs (left AI, right AI) were also obtained from Baur's study (2013). Baur et al. (2013) performed a seed-based rsFC approach to map functionally defined AI based on previous finding that the AI and posterior insula (PI) were respectively connected to the dACC and secondary somatosensory cortex (Cauda et al., 2011). Additionally, we created 6-mm-radius spherical dACC ROIs centered on the respective coordinates (MNI; 5, 26, 31 for right dACC; −5, 26, 31 for left dACC) from the study of Baur et al. (2013) with DPARSF software.

### Association between anxiety and ROI-wise functional connectivity and second level analysis of the functional connectivity

To exclude the effects arising from micro-motion, the “scrubbing” procedure described by Power et al. (2012) was used. The framewise displacement (FD) of head position was calculated as the sum of the absolute values of the six translational and rotational realignment parameters. In the current study, FDs were first computed on a frame-by-frame basis for each participant. Frames with FDs larger than 0.5 were removed from subsequent intrinsic functional connectivity analysis. After this “scrubbing” procedure, an average of 90.9% (7.6% standard deviation) of the frames remained. In other words, only about 9.1% of the data were removed from the statistical analysis. With DPARSF, for each BLA, AI, and dACC subregion ROI, mean time courses were extracted from the data after being scrubbed using the before-described procedure and cross-correlated. Next, correlations were *r*-to-*z* transformed for group-level statistics. Firstly, we examined whether the connections (*z*-value) between six nodes in the SN exist by using one sample *T*-test. Secondly, we used Pearson correlations to investigate whether the rsFC magnitudes between BLA, AI and dACC (*z*-value) were correlated with trait anxiety. All *p*-values were two-tailed. Correlations were evaluated with IBM SPSS16 (IBM, Armonk, New York). Moreover, to examine whether intra-salience network FC are influenced by the participants' emotional states before scanning, we found the Pearson correlation between the NAS and the same rsFC in the SN.

### Voxel-wise functional connectivity analyses and associations with anxiety

We used the same ROIs from the ROI-wise analysis when we extracted time courses to make the voxel-wise functional connectivity analysis. Furthermore, the correlations between the trait anxiety score and the whole brain functional connectivity were calculated with the REST toolbox (Song et al., 2011). Finally, we achieved six related whole brain maps by setting the threshold at a significance level of *p* < 0.05 and correcting for multiple comparisons using the AlphaSim correction in REST. Briefly, the statistical threshold was set at *p* < 0.005 and the cluster size was set at >324 mm^3^, which corresponded to a corrected *p* < 0.05. This correction was confined within whole-brain mask (size: 1912437 mm^3^) and was determined by Monte Carlo simulations (Ledberg et al., 1998) using the AFNI AlphaSim program (http://afni.nimh.nih.gov/pub/dist/doc/program_help/AlphaSim.html). Depression score was entered into the analysis as a covariable, and partial correction results were achieved (AlphaSim corrected *p* < 0.05).

## Results

### Emotional measurements

Emotional measurements of the remaining 60 participants (15.68 ± 1.00 years, 35 Male/25 Female, 15.65 ± 1.01 years for female, 15.73 ± 1.00 years for male) were acquired see Table 1 for TAI and CDI scores in the sample.

**Table 1:** **Demographics and emotional measurements**.

	**M ± SD (*N* = 60)**
Gender(n: male/female)	35M/25F
Age(years)	15.68 ± 1.00
TAI	39.63 ± 6.12
CDI	11.13 ± 2.65
NAS	19.98 ± 5.01

### Correlations between intra-network functional connectivity of the SN and anxiety level

**Figure 1:**
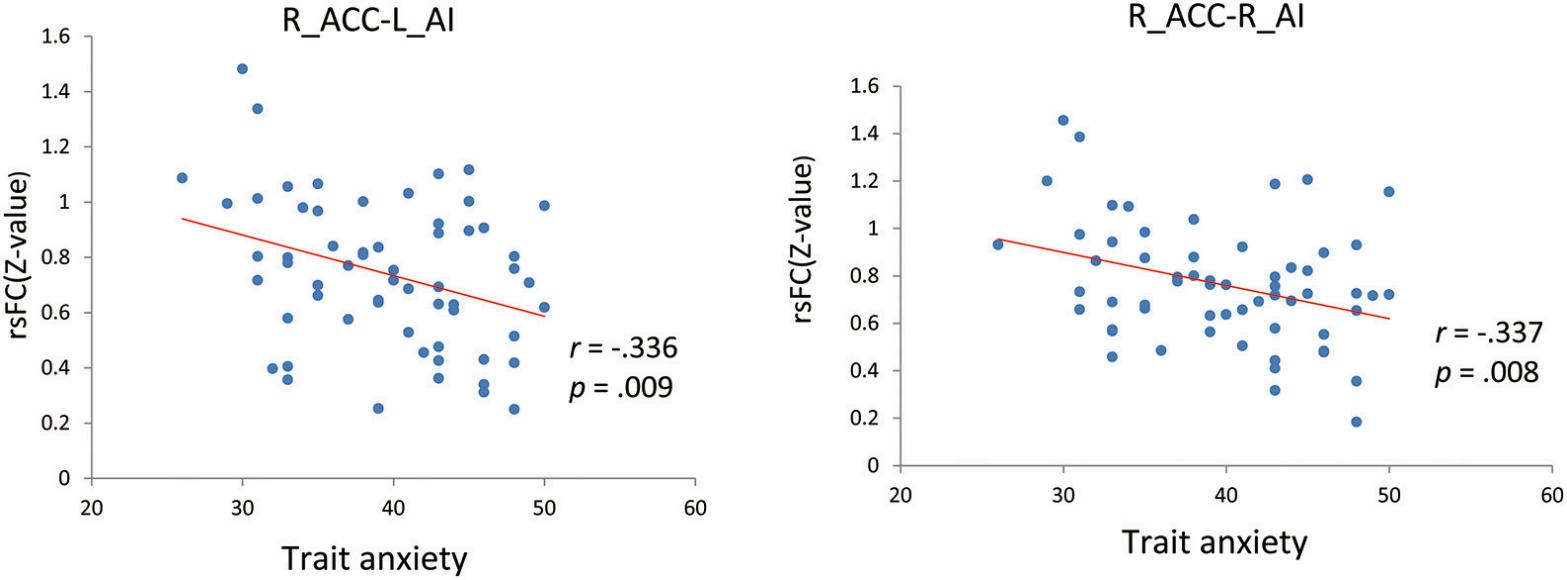
**Correlations of AI-dACC resting-state functional connectivity with trait anxiety**. L, Left; R, Right; ACC, anterior cingulate cortex; AI, anterior insula; rsFC, resting-state functional connectivity.

We firstly examined the 12 functional connections between the six nodes in the SN. The finding showed that all 12 connections were significantly more than zero (shown in Supplementary Table 1 in the Supplemental Material). Furthermore, we tested the correlation between BLA, AI, and dACC rsFC (left/right sides) and trait anxiety in the whole sample. Significant negative correlations were observed between the left AI-right dACC, right AI-right dACC FC and trait anxiety (uncorrected:*r* = −0.336, *p* = 0.009; *r* = −0.337, *p* = 0.008; Figure 1). In addition, depression and trait anxiety were significantly correlated (*r* = 0.525, *p* < 0.001); after controlling for depression, the left AI-right dACC and right AI-right dACC FC correlations with anxiety were marginally significant (*r* = −0.219, *p* = 0.095; *r* = −0.224, *p* = 0.087). Furthermore, no correlation was observed between the AI-BLA or dACC-BLA functional connections and anxiety or depression. Additionally, the NAS score was correlated with trait anxiety (*r* = 0.333, *p* = 0.009), and no correlation between the FC in the SN and the NAS was found (Table 2).

**Table 2:** **Correlations of intra-salience network FC with NAS**.

**Resting-state connectivity**	**NAS**	
	***r***	***p***
left AI-left BLA	−0.031	0.811
left AI−right BLA	−0.096	0.463
left AI−left dACC	−0.108	0.409
left AI−right dACC	−0.138	0.291
right AI−left BLA	−0.069	0.599
right AI−right BLA	−0.081	0.541
right AI−left dACC	−0.088	0.502
right AI−right dACC	−0.072	0.583
left BLA−left dACC	0.062	0.634
left BLA−right dACC	0.003	0.981
righ BLA−left dACC	−0.014	0.913
right BLA−right dACC	−0.034	0.793

### Correlations between inter-network functional connectivity of the SN and anxiety level

**Figure 2:**
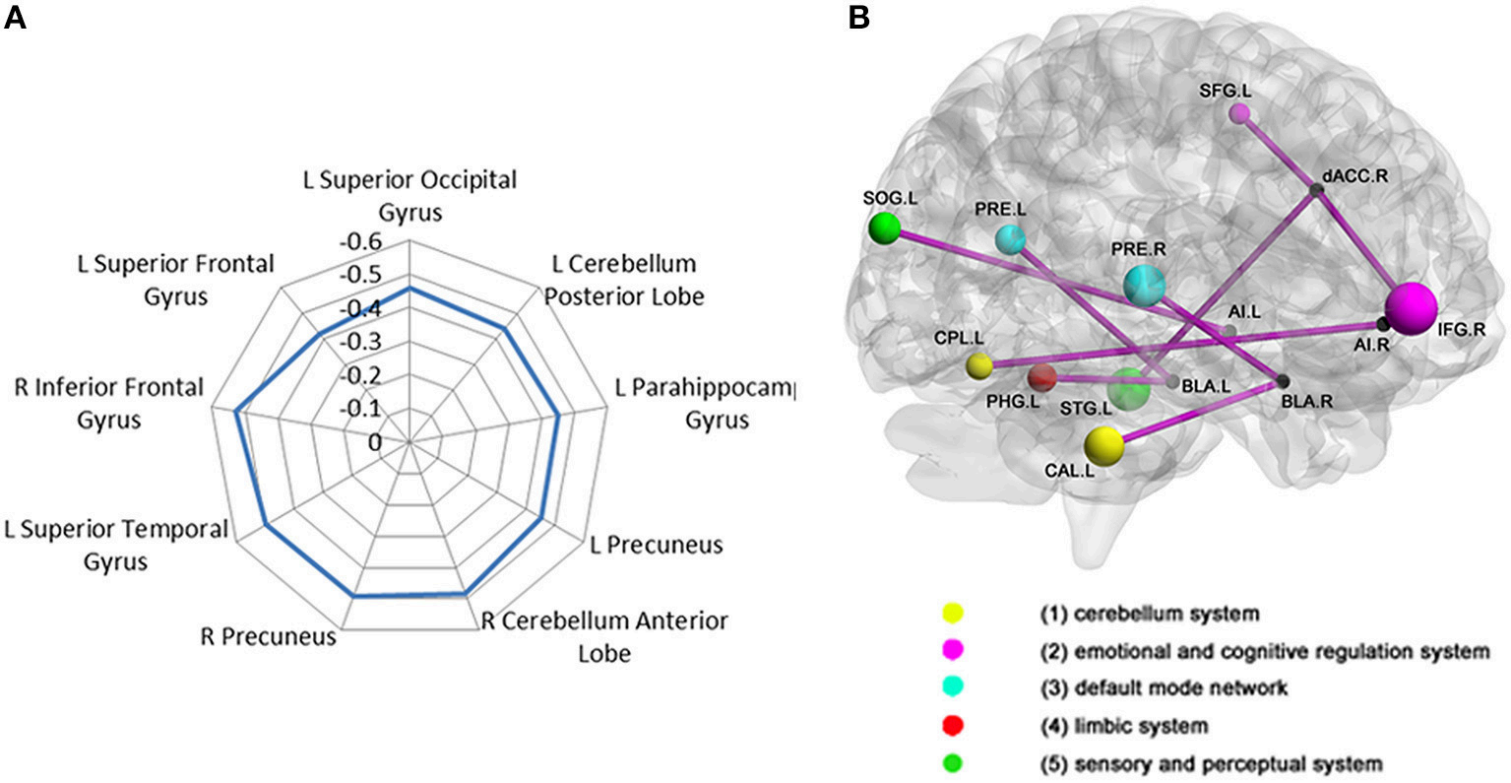
**Correlation between adolescent anxiety and inter-SN functional connectivity**. **(A)**Radar plot showing the correlation between anxiety and inter-salience network connectivity with multiple regions broadly classified into five functional brain systems: (1) cerebellum system (2) emotional and cognitive regulation system (3) DMN (4) limbic system (5) sensory and perceptual system. **(B)** Brain regions (represented by different color-coded nodes) that showed a significant correlation between anxiety and inter-salience network functional connectivity. CAL, CerebellumAnteriorLobe; CPL, CerebellumPosteriorLobe; IFG, InferiorFrontalGyrust; SFG, SuperiorFrontalGyrus; PRE, precuneuns; PHPG, parahippocampalgyrus; IOG, InferiorOccipitalGyrus; STG, SuperiorTemporalGyrus; L, left; R, right.

The voxel-wise FC analysis showed that higher levels of trait anxiety in adolescents were related to weaker functional connectivity of the SN with a number of brain systems outside this network (Figure 2, Table 3, Supplementary Figures 1–6 in the Supplemental Material). The regions involved included the following: (1) the left and right precuneus, which are part of the DMN, (2) the superior temporal gyrus, superior occipital gyrus and fusiform in the sensory and perceptual processing system, (3) the limbic (parahippocampal gyrus) and cerebellum system, including the pons and declive, and (4) the emotional and cognitive regulation regions, including the inferior frontal gyrus and the superior frontal gyrus. There was no region that showed a larger connectivity with the SN. When depression was entered in the analysis of the whole brain functional connectivity, control analyses revealed that the findings above remained seldom changed in the whole brain (Table 4).

**Table 3:** **Results of correlation of two sides of BLA, AI, and dACC voxel-wise functional connectivity with trait anxiety**.

**Region of interest**	**Region**	**Cluster size (# voxels)**	**Maximum intensity voxel coordinates**			***r*-value**
**LEFT BLA**						
	L Middle Temporal Gyrus	13	−51	−15	−18	−0.561
	L Parahippocampa Gyrus	13	−36	−33	−18	−0.479
	L Precuneus	64	−12	−60	18	−0.468
**RIGHT BLA**						
	R Cerebellum Anterior Lobe	69	18	−51	−36	−0.479
	L Cerebellum Posterior Lobe	17	−21	−78	−30	−0.425
	R Precuneus	32	15	−36	6	−0.446
**LEFT AI**						
	L Cerebellum Posterior Lobe	27	−24	−84	−33	−0.444
	R Cerebellum Posterior Lobe	13	27	−81	−33	−0.454
	L Fusiform	29	−36	−57	−21	−0.488
	R Cerebellum Anterior Lobe	17	6	−66	−12	−0.437
	L Occipital Lobe	15	−30	−75	−6	−0.476
	R Inferior Frontal Gyrus	12	57	30	9	−0.426
	L Superior Occipital Gyrus	16	−18	−96	21	−0.453
	R Cingulate Gyrus	17	15	−15	39	−0.495
	R Middle Frontal Gyrus	33	36	−3	48	−0.486
	R Parietal Lobe	13	51	−18	60	−0.444
**RIGHT AI**						
	L Cerebellum Posterior Lobe	22	−9	−72	−15	−0.478
	R Anterior Cingulate	25	12	27	27	−0.458
	R Cingulate Gyrus	16	15	6	39	−0.510
**Left dACC**						
	R Cingulate Gyrus	12	12	9	36	−0.458
	L Frontal Lobe	15	−18	−21	36	−0.426
	R Postcentral Gyrus	13	54	−18	57	−0.436
**RIGHT DACC**						
	L Superior Temporal Gyrus	31	−48	3	−21	−0.471
	R Insula	14	33	9	0	−0.431
	R Inferior Frontal Gyrus	70	51	24	0	−0.536
	R Cingulate Gyrus	29	15	12	33	−0.499
	L Superior Temporal Gyrus	14	−42	−27	6	−0.470
	L Superior Frontal Gyrus	13	−6	9	51	−0.449

**Table 4:** **Voxel-wise functional connectivity in the whole brain correlation with anxiety controlling cdi as covariable**.

**Region of interest**	**Region**	**Cluster size (# voxels)**	**Maximum intensity voxel coordinates**			***r*-value**
**LEFT BLA**	**None**					
**RIGHT BLA**						
	L Cerebellum Posterior Lobe	13	−24	−78	−30	−0.457
	R Thalamus	13	21	−15	15	−0.465
**LEFT AI**						
	L Cerebellum Posterior Lobe	25	−24	−81	−30	−0.486
	L Medial Frontal Gyrus	13	−3	51	0	−0.432
	L L Superior Occipital Gyrus	13	−21	−93	24	−0.414
	L Superior Frontal Gyrus	12	−12	54	36	−0.401
	L Middle Frontal Gyrus	13	−42	−3	45	−0.422
	R Middle Frontal Gyrus	12	36	0	45	−0.446
**RIGHT AI**						
	L Medial Frontal Gyrus	14	−9	51	3	−0.409
**LEFT dACC**						
	R Cingulate Gyrus	18	15	15	33	−0.531
**RIGHT dACC**						
	R Inferior Frontal Gyrus	15	51	24	0	−0.475
	R Cingulate Gyrus	13	15	15	30	−0.461
	L Superior Frontal Gyrus	16	−15	57	33	−0.466

*MNI, Montreal Neurologic Institute, coordinates of most significant voxels in cluster; L, left; R, right*.

## Discussion

By linking the intra- and inter-salience network functional connectivity to trait anxiety in adolescents, we found that the intra-network FC of the SN, particularly the AI-dACC circuit, was associated with trait anxiety in adolescents. This pathway has been suggested to play a critical role in cognitive control according to previous studies (Sridharan et al., 2008; Uddin et al., 2011). Furthermore, we found that the distributed inter-network FC between the SN and multiple brain systems (including DMN and CEN) were also related to a vulnerability to anxiety in adolescents. In short, the current study provides direct evidence of resting-state functional connectivity and how it aids in the understanding of the relationship between intra- and inter-network connections of the SN and trait components of vulnerability to anxiety in adolescents.

We found that decreased AI-dACC FC in the SN was associated with higher anxiety in adolescents, which may indicate the altered cognitive control function of the SN in anxiety. This issue has been highlighted by other studies in adults (Menon and Uddin, 2010). Previous studies have revealed that there are functional and structural connections between the two regions (Critchley et al., 2004), which provide a rapid relay of information between the distributed brain systems (Allman et al., 2010). Menon (2011) suggest that this feature of this circuit is fundamental for detecting salience signals and triggering executive control, both of which play central roles in the pathology of anxiety. Our finding extends previous knowledge and indicates that the AI-dACC FC also contributes to trait anxiety in adolescents. Specifically, we found that the connectivity of this neural circuit was negatively correlated with anxiety characteristics. Our result provides initial and direct evidence for understanding the relationship between this pathway in the SN and the anxiety disposition in adolescents. A previous resting-state and structural fMRI study indicated that weaker connections in SN might underlie less flexible cognitive control during childhood compared to adulthood (Uddin et al., 2011). In the present study, the decreased AI-dACC FC in anxious adolescents may be associated with weaker cognitive control, which is consistent with an anxiety theory that suggests that trait anxiety includes an impoverished recruitment of prefrontal attentional mechanisms to trigger the allocation of attentional resource (Bishop, 2009). This model is supported by the discovery that weaker AI-ACC connections in adults were associated with general social anxiety disorder, which indicates problems in attention control and emotion regulation (Klumpp et al., 2012). It is also in line with another study that reported a weaker correlation between the ventromedial prefrontal cortex (including Brodmann 32, dACC) and the bilateral insula, which represents a weaker control function in anxiety-prone adults (Stein et al., 2007). Our finding about the association between the AI-dACC circuit and trait anxiety in adolescents encourages further research into the roles of the SN in the cognitive control involved in the pathology of anxiety.

In the current study, both the BLA-AI and BLA-dACC FC were found in adolescents, but neither the BLA-AI nor the BLA-dACC circuit showed any association with trait anxiety or state emotional level when measured by the NAS in adolescents. However, two recent resting-state fMRI studies in adults found that these two circuits were positively correlated with state anxiety (Kim et al., 2011; Baur et al., 2013). In those studies, the BLA-AI and BLA-AI FC were suggested to underlie the temporal anxiety state. This type of state anxiety reflects a larger sensitivity to salient events, which may be related to the MRI environment considering an MRI scanner can function as a stressor. Conversely, anxiety proneness as a trait, which the current study focuses on, may rely more on the ability to recruit the prefrontal cortex, which is strongly associated with the AI-dACC circuit rather than the BLA-related circuits. In addition, unexpectedly, a correlation between the BLA-AI or the BLA-dACC and the NAS was not found, possibly because the NAS was not sensitive enough to the anxiety state in specific situations, so it could not capture the immediate stressful feeling of an MRI environment. In future studies, we suggest that biological measurements (such as skin conductance response) be used to illustrate the relationship between state anxiety levels and BLA-related connections.

Aside from intra-network connectivity, we also found that decreased connectivity between the key nodes in the SN and the distributed brain areas was associated with higher trait anxiety. These regions can be grouped into several functional systems, which are involved in salience processing and cognitive control (Uddin et al., 2011), as follows: (1) the sensory and perceptual processing network including the fusiform, occipital lobe, and temporal lobe; (2) the DMN including the left and right precuneus; (3) the cerebellum system and limbic system including the parahippocampal gyrus; (4) the emotional and cognitive control system including the inferior frontal gyrus and superior frontal gyrus. Studies in healthy adults with high anxiety and patients who suffered from anxiety disorders have found an abnormality in the connectivity between key nodes in the SN and these systems (Etkin et al., 2009; Liao et al., 2010a,b; Kim et al., 2011). Below, we discuss the potential relationships between these systems and trait anxiety in a point-by-point manner.

First, the functional connectivity between the SN and sensory and perceptual networks decreased as a function of trait anxiety, which is consistent with the resting-state study that found that the adult patients with social anxiety disorder showed weaker FC in somato-motor and visual networks. The weaker connection may be explained by the vigilance and alertness involved in anxiety (Liao et al., 2010a).

Additionally, the SN, particularly the AI, plays a pivot role in switching between the CEN and DMN across task paradigms and stimulus modalities (Menon and Uddin, 2010). Our result is in line with the resting-state study, which found a correlation between insula-DMN FC and self-report anxiety in youths (Dennis et al., 2011). Seeing that the DMN underlies the representation of negative and self-referential information in anxiety and depression (Dennis et al., 2011), the connection between the SN and DMN may help allocate cognitive sources to self-related information processing during the task state, which is highly involved in anxiety (Menon and Uddin, 2010).

The parahippocampal gyrus within the limbic system plays an important role in the encoding and recognition of environmental scenes and faces (Aguirre et al., 1998). The SN-parahippocampal gyrus connection may be related to attentiveness to face and environmental stimuli, but this requires verification from future studies. Additionally, weaker functional connections between the SN and the cerebellum were found in highly anxious adolescents. This result is in line with a previous resting-state study, which found weaker FC in adolescents with GAD compared with the healthy controls (Roy et al., 2013). The SN and cerebellum are involved in detecting errors and conflicts to adjust future performance (Dosenbach et al., 2006; Buckner, 2013). Given that errors and conflicts are both perceived as threats along the dimension of biological salience (Etkin et al., 2011), we suggest that the SN-cerebellum FC may be associated with bottom–up salience detection of errors and conflicts. Briefly, adolescents experience huge changes in social context when salient stimuli and events are involved (Crone and Dahl, 2012). Our results, combined with previous resting-state fMRI studies of anxious individuals, suggest that multiple SN-related functional connections may be involved in salience processing in this particular period.

Finally, decreased functional connections between the SN and both the superior frontal gyrus and the inferior frontal gyrus were observed. Our results may be implied by the resting-state study of adults with a panic disorder, which found weaker FC between the dACC and both the bilateral frontal pole and the right superior parietal lobule (Pannekoek et al., 2013). Those connections have been postulated to have core roles in salience signal transmission to the CEN for cognitive control (Sylvester et al., 2012). The negative correlation between those functional connections and trait anxiety may be related to the weak cognitive control in anxiety, which is supported by the following studies. As Campbell-Sills et al. (2011) suggested, emotionally salient stimuli are more difficult for anxious individuals to engage effective emotional control. Furthermore, Bishop (2007) argued that the ability to recruit the prefrontal control mechanisms should be seen as the key locus of anxiety-related individual differences. Crone and Dahl (2012) suggest that the degree to which cognitive control is engaged in adolescence is strongly influenced by the motivational salience of the context. These arguments are highly consistent with the model that proposes that the SN-centered connection profile detects salience and triggers cognitive control, which plays a key role in anxiety (Menon and Uddin, 2010). The convergence of the current results and previous models suggests that the attenuated inter-network connectivity of the SN is associated with a vulnerability to anxiety in adolescents, which may underlie the salience-related engagement of cognitive control.

Adolescents experience rapid cognitive and emotional changes compared to adults (Crone and Dahl, 2012). The interaction between the environment and the development of the brain may lead to dysfunctions of emotional processing and cognitive control (Paus et al., 2008). Our study makes an important first step in depicting the association between SN-centered brain networks and trait anxiety in adolescents, and it provides some reasonable explanations for the association, which need to be verified by future studies that illustrate the neurocognitive mechanisms underlying these links. Furthermore, longitudinal studies would be necessary to examine the causal correlation between trait anxiety and brain network development.

A few limitations of the current study should be addressed. First, the NAS scale was used to assess the participants' instant emotional states, which was different from other studies that used self-report or the STAI state anxiety scale. Thus, we could not determine the roles of the amygdala-AI and amygdala-dACC rsFC in state anxiety, which have been reported in other resting-state studies (Kim et al., 2011; Baur et al., 2013). Second, although trait anxiety is often used as an index for vulnerability to an anxiety disorder, and high trait anxiety shares common psychological and neural factors with all anxiety disorders, it would be better to directly choose clinical individuals to investigate the role of the SN on the pathology of anxiety disorders. Thirdly, this study only used resting-state MRI data, and it remains unclear whether the SN would also show abnormal intra- and inter-SN connectivity when highly anxious individuals were participating in cognitive tasks that involved salience processing and cognitive control. Thus, our interpretation of the current findings is only supposed and needs future studies to be clarified.

## Conclusion

In the present study, we found the association between trait anxiety in adolescents and characteristic intra-SN (AI-dACC circuit) and distributed inter-SN connections, which are considered to play important roles in salience processing and cognitive control. Our preliminary findings provide implications for and encourage further understanding of the neural network profile involved in the engagement of cognitive control modulated by emotional salience, which has been proposed as the core mechanism underlying anxiety in adolescents.

### Conflict of interest statement

The authors declare that the research was conducted in the absence of any commercial or financial relationships that could be construed as a potential conflict of interest.
