# Supplementary material for: Decreased Intra- and Inter-Salience Network Functional Connectivity is Related to Trait Anxiety in Adolescents
Source: Front Behav Neurosci. 2016 Jan 21;9:350. doi: 10.3389/fnbeh.2015.00350 (PMC4720749; doi:10.3389/fnbeh.2015.00350)
Supplement: Supplementary file 1 [file DataSheet1.docx]

**Results**

**
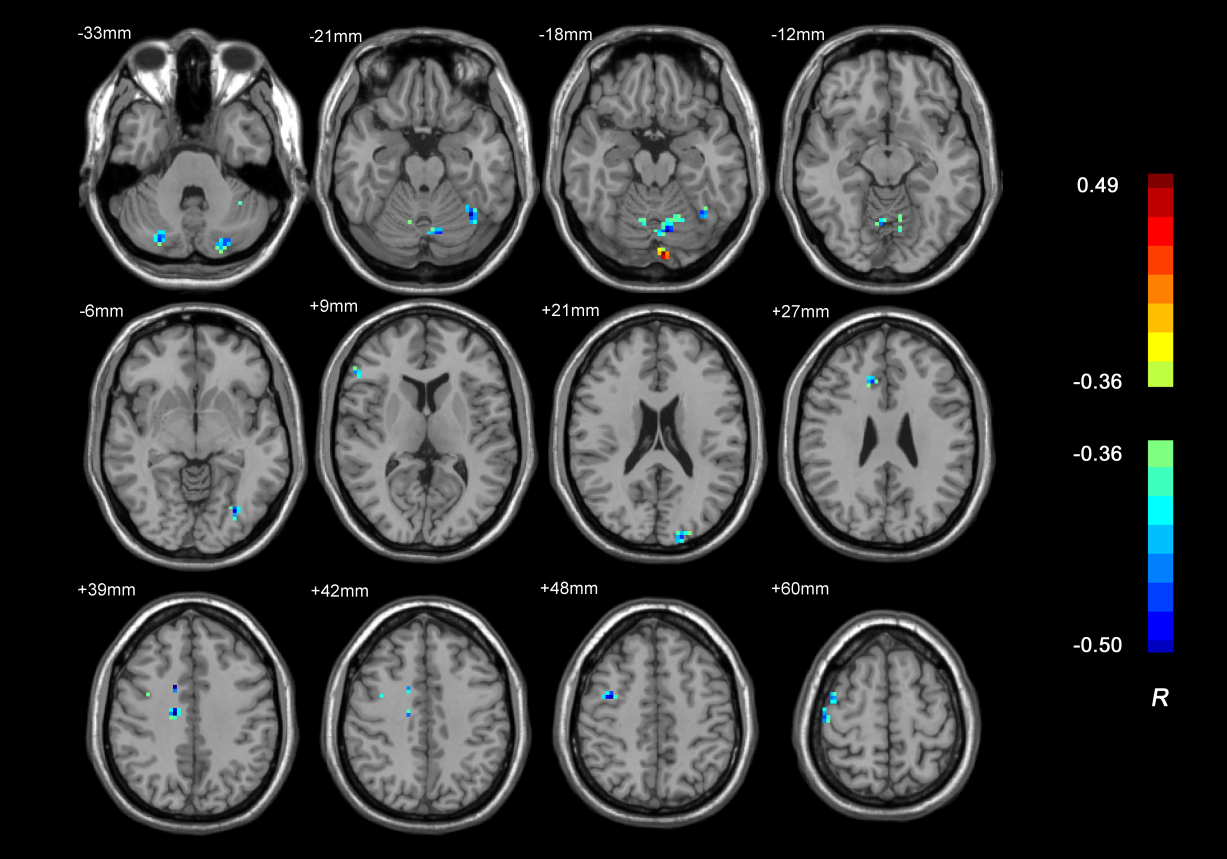
**

Figure 1 Results of correlation of left AI voxel-wise functional connectivity with trait anxiety, AlphaSim-corrected at p < .05


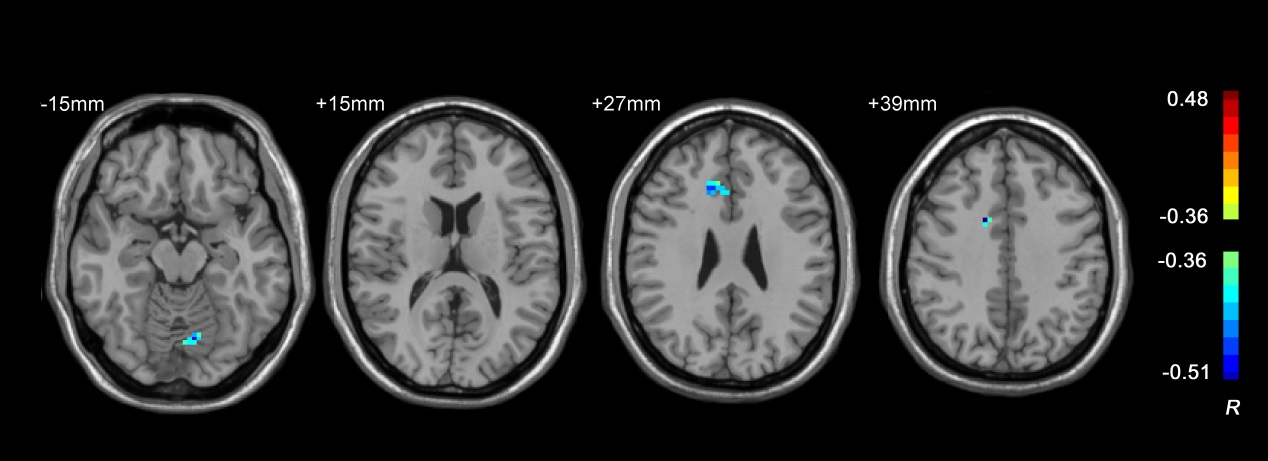


Figure 2 Results of correlation of right AI voxel-wise functional connectivity with trait anxiety, AlphaSim-corrected at p < .05


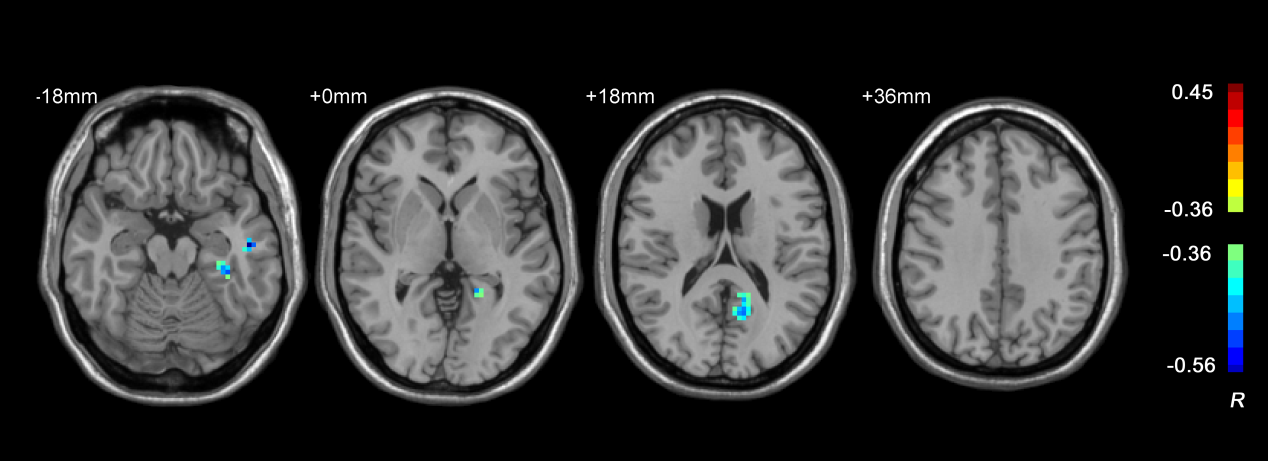


Figure 3 Results of correlation of left BLA voxel-wise functional connectivity with trait anxiety, AlphaSim-corrected at p < .05


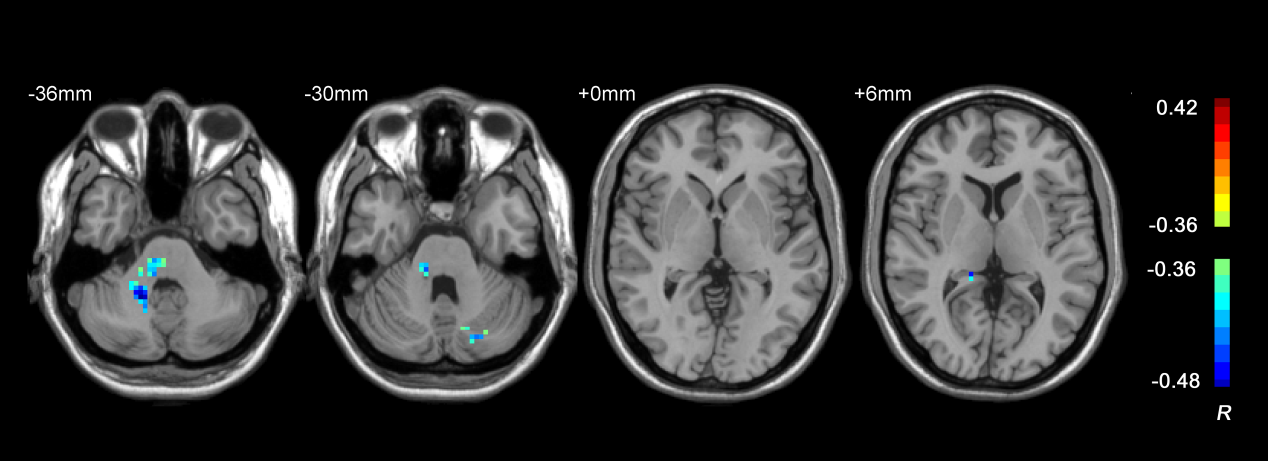


Figure 4 Results of correlation of right BLA voxel-wise functional connectivity with trait anxiety, AlphaSim-corrected at p < .05


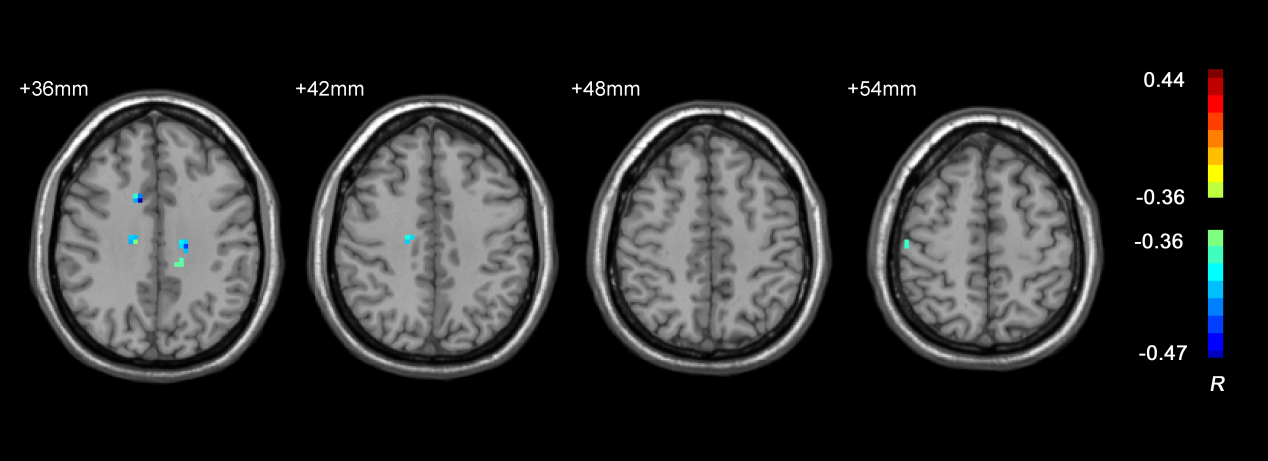


Figure 5 Results of correlation of left dACC voxel-wise functional connectivity with trait anxiety, AlphaSim-corrected at p < .05


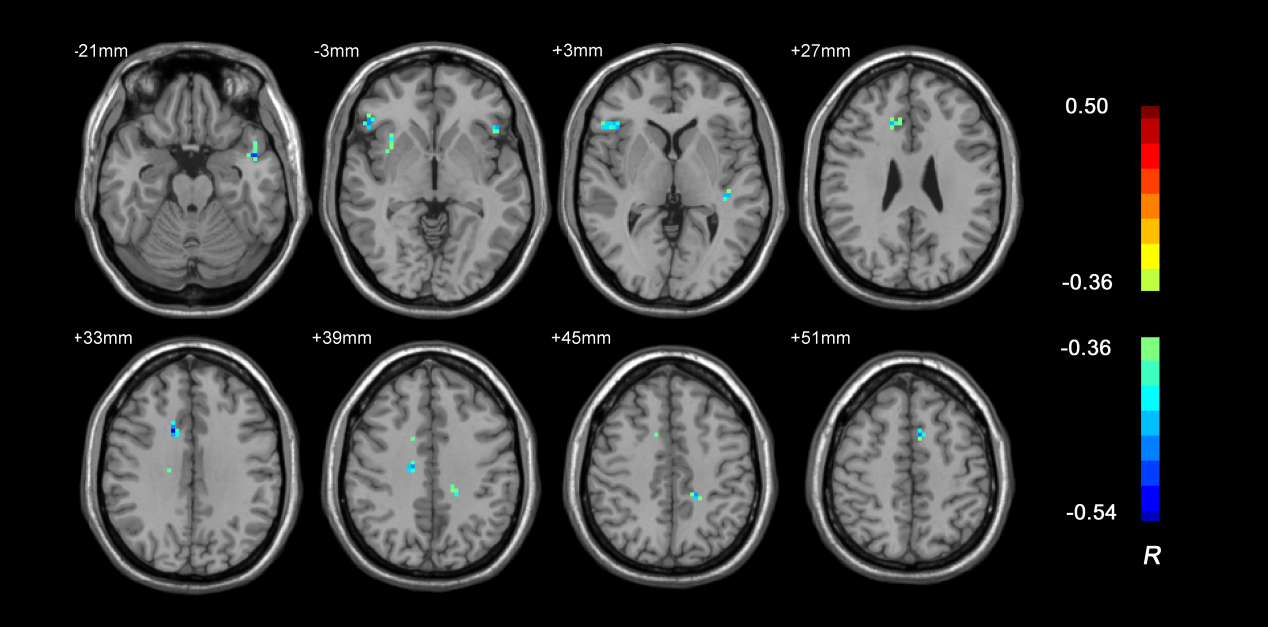


Figure 6 Results of correlation of right dACC voxel-wise functional connectivity with trait anxiety, AlphaSim-corrected at p < .05

Table 1 Intra-salience network connectivity (z-value)

|  | One sample T test | |  |
| --- | --- | --- | --- |
| Resting-state connectivity | *T* | *p* | *Mean(SD)* |
| left AI-left BLA | 6.8047 | 5.786e-09 | 0.25(0.29) |
| left AI-right BLA | 6.5057 | 1.847e-08 | 0.24(0.29) |
| left AI-left dACC | 27.4034 | <2.2e-16 | 0.85(0.24) |
| left AI-right dACC | 21.4027 | <2.2e-16 | 0.74(0.27) |
| right AI-left BLA | 7.7158 | 1.656e-10 | 0.28(0.28) |
| right AI-right BLA | 8.7179 | 3.382e-12 | 0.32(0.29) |
| right AI-left dACC | 20.2019 | <2.2e-16 | 0.70(0.27) |
| right AI-right dACC | 23.3828 | <2.2e-16 | 0.76(0.25) |
| left BLA-left dACC | 6.8179 | 5.498e-09 | 0.21(0.24) |
| left BLA-right dACC | 6.7157 | 8.179e-09 | 0.23(0.27) |
| righ BLA-left dACC | 6.8843 | 4.245e-09 | 0.19(0.22) |
| right BLA-right dACC | 7.159 | 1.455e-09 | 0.23(0.25) |
